# Piano Plaints

**Published:** 1878-10

**Authors:** Ben. H. Pratt


### THE BISTOURY.

Vol. XIV. ELMIRA, N. Y., OCTOBER, 1878. No. 3.

### Contributions.

### PIANO PLAINTS.

BEN H. PRATT.

That which has become pre-eminently the social bore of the age, is the piano-plague. This generation is afflicted to an alarming extent with a polypianic malady which demands a remedy. The mania for piano playing has gotten such a hold upon society, consumes so much of its time, is the cause of so much extravagance, diverts youth from so much that is useful, makes so many good-for-nothing women and namby-pamby men, bores society to such an outrageous extent, accomplishes so little that is good, and so much that is not even entertainment, begets such wrong ideas of duty to self, to society and the world; in fact, is so utterly devoid of utility, and is such an absorbent of vital energy, that the custom invites protest and plain talk on the part of somebody.

Right here it will be best to introduce some qualifying clauses, lest the above paragraph may not convey the writer's sentiments clearly; for he does not claim that piano music should not be cultivated, or that pianists, who are truly such, should not be encouraged to the farthest limit of their genius, or that the piano should be banished from the concert-room or the parlor or the school house, or that every performer upon that instrument is necessarily a merely ornamental appendage to society, or that all pianism is a bore and a social scourge—by no means.

The custom which somebody should inveigh against, and in no measured terms, is that which compels every family, whose claims to gentility are of the average sort, to make pianists of all its daughters, whether they will or no, and of its sons, if it can do so without creating a domestic mutiny in the household. There are born musicians as there are born poets, as there are born doctors, lawyers, preachers, orators, mechanics, scientists, philosophers, artists, architects, highwaymen, thieves, slaves, idiots. As there are diversities of genius which no human power can control, and bents of talent which no force of circumstances can hinder, so there is no human power that can make musicians out of those who are not divinely gifted with a musical taste. To think that the presence of a grand piano in the parlor, a grand musical professor in the town, and a grand balance at your banker's, can make of your girls even mediocre performers upon a noble instrument, is as fallacious an idea as to believe that you can produce figs from thistles or grapes from thorn bushes.

Music is an inheritance. Without the germs of harmony and concordant sounds within the soul, inborn, innate, no art can make of any man or woman a musician. No height of social position, no magnificence of piano, no wealth of treasure and no brilliance of professorial skill can make even passable pianists out of those "who have no music in themselves and are not moved by concord of sweet sounds." And as one-half of mankind lacks the gift necessary to become proficient in music, fashion, the arbiter of society, makes fools of half our girls, and these drive their friends well nigh to distraction by their outrageous attempts to entertain, without a consciousness of the misery their efforts beget.

The wonder is, that society in the aggregate can so complacently suffer what the individual constituents of that society so invariably and unhesitatingly denounce as an unmitigated bore; that in drawing-room assembled, it will go into an artificial spasm of ecstatic admiration over a bit of wretched, ear-offending, nerve-racking key-pawing, and then privately consign the performer to the Hadesian glades as a punishment for the infliction. Society is so self-sacrificing in no other particular, and one can account for the phenomenon in no other way than upon the principle that we seldom point out the infirmities of our neighbors when we ourselves have an equally conspicuous infirmity, of which we are too conscious to make even an indirect allusion to.

Who of us has not suffered from this ridiculous custom of compelling all girls to "take music lessons?" Who of us, to whom society is at all familiar, has not heard the daughter beg exemption from the hated ordeal at the music stool—seen the tears of genuine suffering flow copiously after an hour's siege in attempts to accomplish those "horrid scales"—felt a keen pity for the task-ridden maiden who must needs thump, thump, thump away her wretched two hours a day in piano practice—pitied the parent who must needs be her own child's tormentor, because society demands that she must be a player or lose caste—endured the thrum of mechanical key-pelting hour after hour and day after day in his home—bemoaned the wasted time, and the sacrifice of so much really useful learning to a useless accomplishment, at least one which a girl with no genius for music could not even convert into a pleasure for her friends, and an acquirement which she will certainly abandon when parental authority ceases—who has not had bitter and anger-provoking experience of this kind? We see young women all around us who are growing up intellectual dwarfs because the time which should be devoted to mental culture is used in attempting to acquire that which they cannot master, and would avail them little if they could. Even the most common elements of an ordinary education are often sacrificed to a showy art, and the woman is made a conspicuous target for the arrows of criticism from the quivers of those far below her in the social scale. How compromising the remark often heard, even though the subject be an accomplished artist, "She's a good player, but she knows nothing but music." Yet in cases of undoubted genius, be it musical or other, we can overlook many deficiencies—for rare attainments, even in a single branch of art, excuse much. Blind Tom was not blamed for being an idiot, and Gottschalk's conceit and foppery were not barriers to his musical success. These couldn't help being musicians, and were nonentities in every other particular. They had the genius, the gift, the innate germs of music within them, and so have countless men and women to whose diviner harmonies we now and then listen enchanted. Of such we are not speaking here, but of the multitude of those who rend the air with execrable combinations of sound—who are not discriminators between noise and harmony—whose efforts, which their friends praise in public and damn in secret, are mere mechanical echoes of others' genius, abortions compared with the productions they imitate.

How piano playing ever acquired such a hold upon society is often regarded as one of the undemonstrated problems of the age, but it is not difficult to arrive at the cause, which has its being in the fact—silly as it may seem—that the measure of a family's gentility is the cost of its pianoforte. Pianos are a sort of social gentilitometer, and indicate very accurately the height or depth of the respectability enjoyed by the household. Hence every family is ambitious to possess this resonant passport to social position, and the fortunate possessor of the magic article then finds that Society, though very much inclined to receive and court its new member, requires something more than the mere possession of the instrument, to wit, somebody to manipulate its polished ivory and evoke a din therefrom, which, however far it be removed from music, shall be something thereto akin, at least. Hence the daughter is summoned and hears the fiat that is either the beginning of her misery or the means of discovering a latent genius—generally the former, when all the ills that musical quackery can inflict upon a suffering community follow in their natural sequence, and we have just such a state of things as exists to-day in every village, town and city of the civilized world, and all arising from the fact that the acknowledged criterion of our social status is the cost and elegance of our piano.

It would seem that after a generation or two of women had grown up, enduring the chagrin of knowing little of music, less of grammar and nothing of household duties, and all because of the gentilitometer that stands in the drawing-room, some less sacrificial standard of social position would be chosen, and one which, if it did no particular good, would militate less disastrously against our youths, and especially our girls. It is getting to be a serious matter when young women are taught to believe that skill in evoking music from a piano is the chief ante-matrimonial end and aim of every maiden, and that society demands only this from them.

We attended, not long since, the commencement day exercises of what, in the usual acceptation of the term, is known as a large, popular, and highly successful Female Seminary. The programme was three hours long, and not in all that time was the audience treated to a single essay, recitation, reading, or other literary exercise. All was music, vocal and instrumental, solos, duos, trios, quartettes, quintettes, sextettes, choruses, and two, four and eight hand gymnastic exercises upon pianos, until one felt as though he were chock full of thump and squeal, and didn't want to hear any more of that sort of thing for a twelvemonth. Now and then, among the performers, was a musical genius—one who loved the divine art, and put her whole soul into her work ; but the majority of the "sweet girl graduates," and undergraduates, were as utterly devoid of the requisites necessary to make even passable musicians, as it was possible for human beings to be. Every movement indicated this. They went to their work with distaste for it plainly imprinted upon every feature. They executed their pieces not only in a manner distressingly mechanical, but in a style so purely imitative that the merest tyro in musical criticism could not fail to see that they were mere victims to the family pride. Such will, during their ante-matrimonial lives, continue to inflict upon their friends all manner of musical atrocities, and then forever abandon the piano upon the plea that household duties demand their undivided attention, and they "get no time for practice." Society, thanks be for that, accepts this plea ; otherwise our lunatic asylums must needs be enlarged.

We give our girls good excuses to be dunces. We demand of them, regardless of their tastes and inclinations, hours upon hours of daily practice at the piano. They may neglect their studies at school—be excused from rendering their mothers any assistance in household affairs—grow up ignorant of cookery and the fashioning of their own garments—be allowed almost any indulgence not absolutely criminal—but "their music" must never be allowed to lapse one jot or tittle. Girls grow up weaklings for want of air and exercise, because of that piano and the maternal determination that our daughters shall show off as well as Smith's. You may talk as much as you like, but you can't persuade the mother of the most unmusical girl in the community that it's no use to give that girl music lessons. You may suggest that whistles cannot be made of pigs' tails, only to be met with the rejoinder that very good whistles *have* been made from porkly extremities, and it's only a question of harder study and more hours of it, to make as good musicians of those who haven't musical genius as of those who have that genius. And here's where the trouble comes. Smith's daughter longs for her piano, and her hours of communion with its grand tones are seasons of ecstacy. Brown's girl hears and envies her, but knows she cannot achieve her excellence any more than a crow can assume the role of a nightingale. But Mrs. Brown, not so fixed ln her notions of innate and acquired gifts, insists that her crow only needs a little more practice and study to make of herself a nightingale, and persists in trying to perform the ornithological miracle. Hence the eternal paw-pawing, and the fearful caw-cawing to which those within ear-shot of Mrs. Brown's piano must submit, and gracefully, for when there are motes in some eyes, behold are there not beams withal, in others ?

If there is any dogma of society into which less common sense enters than in this mania for making pianists of all girls whose fathers are able to purchase a piano, we have failed to find it. Even in matters of dress, and in the thousand social formulæ of the age, there is some adaptation of the mode to the individual. It is not demanded that the blonde shall wear red, and the brunette blue, but that each shall adopt her appropriate color. It is not at all essential to the social standing of the young woman of this day that she shall be versed in the knowledge of French, or German, or of the classics ; but in music she must at least have the ability to show that certain hundreds of dollars have been expended upon her, exclusive of the big thing on carved legs that monopolizes so much of the space in the drawing-room. To demonstrate this has cost more than money, but the sacrifice of health and useful knowledge is, to our shame be it said, never made the source of a spoken regret, notwithstanding that there is a tacit admission of the fact permeating the very society which has allowed itself to become the slave of a silly and exceedingly compromising custom. Hence, as we before remarked, somebody is called upon, in the name of humanity, and in defence of the physical and mental well being of our girls, to protest against this universal attempt to make musicians out of the unmusical, and shock the tuneful sense of the cultivated in an art too closely allied to something divine to be outraged with impunity.

There is a demand for some more sensible evidence of social standing than the possession of a grand piano, and a daughter to pelt and paw it. We might suggest, as a far preferable evidence of ultra respectability, the possession of a white elephant, which the sons might be taught to ride with far more grace, and in a much shorter time, than the daughters can become even poor pianists. The antics of an apt monkey, trained for the entertainment of visitors, would much less shock the nerves of most people than the so-called fantasies of some of our forced pianists. The theme is a suggestive one, and we doubt very much whether the inventive genius of those most fertile in expedients could be employed to better advantage than in devising a substitute for the piano pest.

There is no good reason for the adoption of the piano as the universal test of a certain sort of social eminence. True, it is the grandest of musical instruments, save the pipe organ, but that is no reason why it should be made a source of annoyance to one-half the community. On the contrary, this is just why it should become the medium of conveying pleasure only, and they alone should be trained to use it whose inclinations lead them to a mastery of its powers—who do not make tasks of their hours of practice—who do not beg to be excused when invited to entertain their friends with their skill—who love the instrument rather for the joy it gives them than because of the social position to which its possession has boosted them.

Let our daughters be taught that it is not the *sine qua non* of respectability to possess a piano; or if there is one in the house, that it is not an essential element to their future welfare—matrimonially or otherwise—that they suffer a daily martyrdom in acquiring a passable skill in evoking musical tones therefrom; or that the piano is more than meat, and the music thereof more than raiment; but that to master our mother tongue is more honorable than to execute, however enrapturingly, one of Beethoven's grandest productions; that to lift from her mother's hands the heavier burdens of the household, is a better recommendation than to play the piano however divinely; that the "coming man," if he's worth having, won't place so much stress upon the elegant as upon the practical and useful accomplishments of the sensible maiden of whom he is in search; that the money expended in teaching one, who is not musically endowed, to play very indifferently, would set up a very fair home for a newly wedded pair, even if a little over fastidious as to their luxuries; that after all, not one in twenty wives ever make any use whatever of all the music they have expended years and tears upon, and sacrificed so many pleasures and useful acquirements for; that, in a word, the whole system of making proficiency at the piano a test of social status and matrimonial eligibility, is a delusion and a snare, the which few girls see until they become madams, and madams, strange to say, don't see that what was their bane is going to be the bane of their daughters.

Let some sensible matron, with a position which a dethroned piano couldn't affect, and with daughters minus musical genius—no disgrace, either—try the anti-pianic plan of procedure, for her daughters' sakes. We predict that, other things being equal, they will know more, be better members of society, have better lungs and muscles, possess cheerier dispositions, get better husbands and live to be better mothers and die better christians for the change. Who will do it, and start an era of common sense in society?

